# Spatial synchrony cascades across ecosystem boundaries and up food webs via resource subsidies

**DOI:** 10.1073/pnas.2310052120

**Published:** 2024-01-02

**Authors:** Jonathan A. Walter, Kyle A. Emery, Jenifer E. Dugan, David M. Hubbard, Tom W. Bell, Lawrence W. Sheppard, Vadim A. Karatayev, Kyle C. Cavanaugh, Daniel C. Reuman, Max C. N. Castorani

**Affiliations:** ^a^Department of Environmental Sciences, University of Virginia, Charlottesville, VA 22904; ^b^Center for Watershed Sciences, University of California, Davis, CA 95616; ^c^Department of Geography, University of California, Los Angeles, CA 90095; ^d^Marine Science Institute, University of California, Santa Barbara, CA 93106; ^e^Department of Applied Ocean Physics and Engineering, Woods Hole Oceanographic Institution, Woods Hole, MA 02543; ^f^Marine Biological Association of the United Kingdom, Plymouth PL1 2PB, United Kingdom; ^g^Department of Ecology and Evolutionary Biology and Kansas Biological Survey and Center for Ecological Research, University of Kansas, Lawrence, KS 66047

**Keywords:** spatial synchrony, resource subsidies, giant kelp, wrack, shorebirds

## Abstract

Cross-ecosystem subsidies are critical to ecosystem structure and function, especially in recipient ecosystems where they are the primary source of organic matter to the food web. Subsidies are indicative of processes connecting ecosystems and can couple ecological dynamics across system boundaries. However, the degree to which such flows can induce cross-ecosystem cascades of spatial synchrony, the tendency for system fluctuations to be correlated across locations, is not well understood. Synchrony has destabilizing effects on ecosystems, adding to the importance of understanding spatiotemporal patterns of synchrony transmission. In order to understand whether and how spatial synchrony cascades across the marine-terrestrial boundary via resource subsidies, we studied the relationship between giant kelp forests on rocky nearshore reefs and sandy beach ecosystems that receive resource subsidies in the form of kelp wrack (detritus). We found that synchrony cascades from rocky reefs to sandy beaches, with spatiotemporal patterns mediated by fluctuations in live kelp biomass, wave action, and beach width. Moreover, wrack deposition synchronized local abundances of shorebirds that move among beaches seeking to forage on wrack-associated invertebrates, demonstrating that synchrony due to subsidies propagates across trophic levels in the recipient ecosystem. Synchronizing resource subsidies likely play an underappreciated role in the spatiotemporal structure, functioning, and stability of ecosystems.

## Significance

Many ecosystems depend on resource inputs, called subsidies, from other ecosystems, influencing their structure and function. Using a model system, we demonstrate how resource subsidies can synchronize the dynamics of recipient ecosystems across space: Synchronous offshore kelp supply, mediated by wave action and beach width, yielded synchronous deposition of kelp wrack (detritus) on open coast sandy beaches, which then cascaded through the trophic levels of the terrestrial recipient system, synchronizing local abundances of shorebirds that move among beaches to forage on invertebrate wrack consumers. Cross-ecosystem synchronization via subsidies likely plays a major but previously unrecognized role in the spatiotemporal dynamics and stability of recipient ecosystems.

Flows of matter and energy across ecosystem boundaries form critical links between ecosystems and can dramatically affect the structure and function of donor and recipient ecosystems (1–3). However, the extent to which such flows are synchronized across locations, and whether such flows can induce synchronization in the dynamics of recipient ecosystems, is essentially unknown. Here, synchronization refers specifically to spatial synchrony, the tendency for temporal fluctuations in an ecological variable to be correlated across locations. Spatial synchrony is ubiquitous and manifests in important phenomena including outbreaks of pests and disease (4, 5). Synchrony also has major consequences for ecosystem stability, since synchronous fluctuations reinforce each other when summed, totaling to large regional variations (6). Thus, cascades of spatial synchrony across ecosystem boundaries—wherein spatial synchrony in one kind of ecosystem induces spatial synchrony in a different kind of ecosystem—may potentially account for major regional ecosystem fluctuations, though whether such cascades actually occur in real ecosystems has not previously been examined.

Cross-ecosystem material exchanges are exceedingly common (1) and have important consequences for community structure and dynamics as well as numerous ecosystem functions such as resource provisioning and nutrient cycling (2). Cross-ecosystem flows connect ecosystems within and across terrestrial, freshwater, and marine environments, and their magnitude can equal or exceed local production (1). Consumer population dynamics, species interactions, and food web complexity often differ between systems with consistent in situ production versus those more dependent on subsidies (7, 8), and theory predicts that ecosystems with low in situ resource supply relative to the magnitude of subsidy inputs experience strong trophic cascades in response to subsidies (9). Relatedly, phenomena like mast seeding can create high-amplitude resource pulses, the consequences of which cascade through ecosystem dynamics (8, 10–12). Taken together, these bodies of research demonstrate that variability in the spatial and temporal dynamics of resource subsidies can have dramatic consequences for ecosystem stability (10, 11, 13), though the effects of subsidies on spatial synchrony, which we examine here, were previously unexplored.

This study examines the potential for resource subsidies from kelp forests to induce spatial synchrony in sandy beach ecosystems. Giant kelp (*Macrocystis pyrifera*) is an inter-continentally distributed foundation species (14). Giant kelp is a dominant source of primary production on rocky reefs, creating underwater forests that support a biodiverse reef ecosystem (15, 16) and subsidize adjacent ecosystems through the export of detrital organic material (17, 18). Giant kelp has cultural and socioeconomic value through supporting fisheries and ecotourism (14). Giant kelp forests have also been central to the development of influential concepts in ecology, including foundation species (14, 15), keystone species (14), and trophic cascades (19). Recent studies have discovered that giant kelp biomass on reefs is synchronous across a range of spatial and temporal scales in response to dispersal and climate-driven environmental conditions (20–22).

Kelp forests provide a resource subsidy to sandy beaches in the form of kelp wrack (i.e., detrital kelp biomass). Kelp wrack is a basal resource in multitrophic beach food webs (23), where wrack propagates as energy throughout the food web from primary consumers including microbes and detritivores (17, 18) to mammals and shorebirds (24, 25). Similar to kelp forests’ conceptual importance in ecology, beaches have been central to understanding resource subsidies (26). Coupled systems, such as these, which involve uni-directional subsidies to an ecosystem with low in situ productivity, are ideal for exploring cross-ecosystem synchrony because of the relative ease of quantifying the magnitude of subsidies and identifying their effects on the recipient ecosystem.

Using kelp forests and sandy beaches as a model system, this study examines whether and how resource subsidies can synchronize the dynamics of recipient ecosystems. Specifically, we ask: 1) How spatially synchronous are resource subsidies, here taking the form of kelp wrack deposited on sandy beaches, and can synchronous resource subsidy magnitudes in the recipient ecosystem be attributed to synchronous production of the resource in the donor ecosystem, transmitted across ecosystem boundaries? 2) Does synchrony in the resource subsidy propagate through the recipient ecosystem across trophic levels, here exemplified by the observed local abundances of shorebirds that feed on wrack-associated invertebrates? We show that resource subsidies cause spatial synchrony to cascade across ecosystem boundaries and have multitrophic effects on recipient systems. Ecosystem synchronization via resource subsidies is likely a widespread but underappreciated phenomenon due to the commonness of its key constituents, with significant implications for the stability of both donor and recipient ecosystems.

## Results

**Fig. 1.**
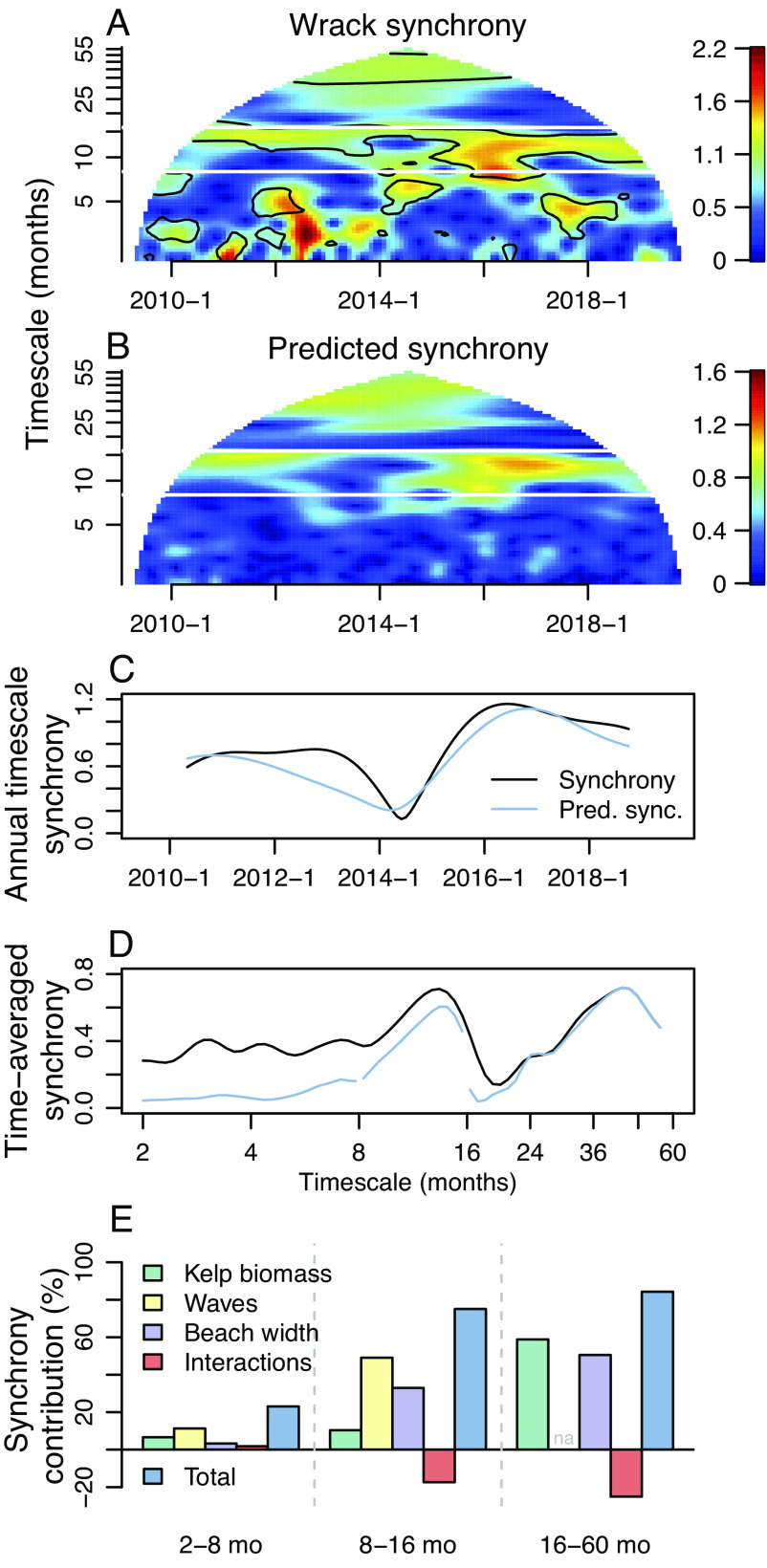
Spatial synchrony in kelp wrack is explained by synchrony in live kelp biomass, waves, and beach width. (*A*) Time and timescale-specific synchrony in kelp wrack depicted by the wavelet mean field. Black contours indicate statistically significant synchrony as determined from the wavelet phasor mean field. (*B*) Wavelet-linear-model-predicted wrack synchrony resembles empirical wrack synchrony on approximately annual (8 to 16 mo) and interannual (16 to 60 mo) timescale bands, but not on the intra-annual band (2 to 8 mo). Note that the three bands were modeled separately. (*C*) Isolating the wavelet component at ≈12 mo, predicted synchrony tracks observed synchrony through time. (*D*) The models explain substantial fractions of time-averaged spatial synchrony, especially at 8 to 16 mo and 16 to 60 mo timescales. (*E*) Fractions of synchrony in beach wrack explained by live kelp biomass, waves, beach width, and two-way interaction effects. Interaction effects can be positive (synergistic) or negative (antagonistic). Waves were not included in the 16 to 60 mo timescale model due to lack of significant coherence at these timescales (Table 1).

Fluctuations in the abundance of kelp wrack were spatially synchronous among five sandy beaches near Santa Barbara, California, USA, during the 11-y study period (Fig. 1*A*). We used the wavelet mean field (27) to uncover time- and timescale-specific patterns of spatial synchrony in giant kelp wrack and the wavelet phasor mean field (27) to ascribe statistical significance (Methods). We observed significant spatial synchrony (black-outlined regions in Fig. 1*A*) episodically at 2 to 8 mo timescales and more consistently at 8 to 16 mo and at some timescales beyond 16 mo. We then focused on three timescale bands, which encompass annual seasonal cycles (8 to 16 mo) and all shorter (i.e., intra-annual, 2 to 8 mo) and longer (i.e., interannual, 16 to 60 mo) timescales that can be resolved in the data.

Kelp wrack synchrony was largely explained by synchronous fluctuations in live kelp biomass and wave action, demonstrating the transmission of synchrony across the marine-terrestrial boundary, and also by fluctuations in beach width (Fig. 1 *B*–*D*). Here, wave dynamics are represented by the first difference of monthly wave heights so that positive values correspond to periods where wave action is increasing. Change in wave action, as opposed to wave action itself, is key to wrack dynamics because as waves fragment and dislodge kelp to produce wrack, less kelp remains to be removed and progressively more wave energy is required to produce more wrack (28, 29). For example, if two equally intense storms occur in quick succession, waves from the first storm will generally remove more kelp than the second. Some amount of dry beach must be present for wrack to be retained and as dry beach width increases so does the potential amount of wrack that can be deposited (30–32).

We constructed wavelet linear models (33) for each focal timescale band using a combination of candidate predictors (live kelp biomass, waves, and beach width) that were individually coherent (spatial wavelet coherence P<0.1) (27) with kelp wrack at that timescale band (Table 1). Live kelp biomass was considered at local and region-wide spatial scales using satellite data (34, 35), and when both were coherent with wrack, we selected the scale with the smaller P-value for inclusion in the multivariate model. At the 2 to 8 mo and 8 to 16 mo timescale bands, synchrony in kelp wrack was explained by the combination of regional kelp biomass, waves, and beach width. At 16 to 60 mo timescales, synchrony in kelp wrack was explained by regional kelp biomass and beach width, but not by waves. These predictors and their interactions explained a modest fraction of wrack synchrony at 2 to 8 mo timescales, and substantial fractions of wrack synchrony at 8 to 16 mo and 16 to 60 mo timescales (Fig. 1). Interaction effects between predictors can, in general, be either positive (i.e., synergistic—wrack synchrony was enhanced by the combination of both effects, above the expectation if both effects acted independently) or negative (antagonistic—wrack synchrony is reduced by the combined effects of both variables), though interactions were negligible or negative for our models of wrack synchrony (Fig. 1*E*). Interactions between Moran effects are a relatively new idea, but have been well documented (21, 33); and a general theory of Moran interactions has been developed (36).

**Table 1.** Relationships between kelp wrack and driver variables

Driver variable	2 to 8 mo	8 to 16 mo	16 to 60 mo
Local kelp	P=0.695	P=0.262	P=0.081
biomass			
Regional kelp	P=0.086	P=0.010	P=0.002
biomass	ϕ=−0.76	ϕ=−0.18	ϕ=−0.77
Waves	P=0.017	P=0.002	P=0.317
	ϕ=0.37	ϕ=0.17	
Beach width	P=0.013	P=0.004	P=0.047
	ϕ=−0.19	ϕ=0.01	ϕ=−0.12

P-values are from tests of spatial wavelet coherence; coherent drivers were selected for multivariate wavelet linear models. Phase relationships (*ϕ*) for selected variables (*Materials and Methods*) were obtained from multi-predictor wavelet linear models. Phase relationships are given in fractions of *π*. Negative phase relationships that are not approximately in-phase (ϕ≈0, interpreted as −0.25<ϕ<0.25) or anti-phase (ϕ≈±1, interpreted as ϕ<−0.75 or ϕ>0.75) indicate that wrack lags the driver variable; positive phase relationships that are not approximately in-phase or anti-phase indicate that wrack peaks precede those of the driver variable.

Phase relationships (ϕ) between kelp wrack and its drivers (Table 1) shed light on the ecological mechanisms by which synchrony is transmitted across the marine-terrestrial boundary, from the kelp forest to the beach. At 8 to 16 mo timescales, encompassing annual cycles, relationships between live kelp biomass, waves, and beach width were approximately in-phase (positive associations), consistent with the hypothesized mechanistic effects of each driver. Large standing biomass of giant kelp (high density covering a large offshore area) was associated with high cover of kelp wrack on beaches. Large waves dislodge and break apart kelp plants, increasing detrital production and beach wrack. Wider beaches provide more area for wrack deposition and retention. Phase relationships varied by timescale, however. At 2 to 8 mo timescales, wrack lagged kelp biomass. Kelp must grow before it can become detritus, whether due to senescence or disturbance, and the associated lag was noticeable on 2 to 8 mo timescales but negligible over longer timescales. Similarly, peaks in wrack tended to slightly precede peaks in wave height first differences. Kelp plants are likely damaged or uprooted before the strongest waves reach them (28, 29) because kelp are damaged as waves increase prior to peak wave periods. At 16 to 60 mo timescales, encompassing interannual variability, the relationship between wrack and live kelp biomass counterintuitively approached an antiphase (negative) relationship. A likely explanation is that on interannual timescales some factors that promote accumulation of kelp biomass are unfavorable for wrack deposition. Kelp biomass tends to be greatest in years of low storm activity and high seawater nutrient concentrations (21, 37). Although waves were not coherent with wrack on 16 to 60 mo timescales (Table 1), statistical power inherently declines at longer timescales, and statistical detection may be less likely if the primary mechanism is indirect.

**Fig. 2.**
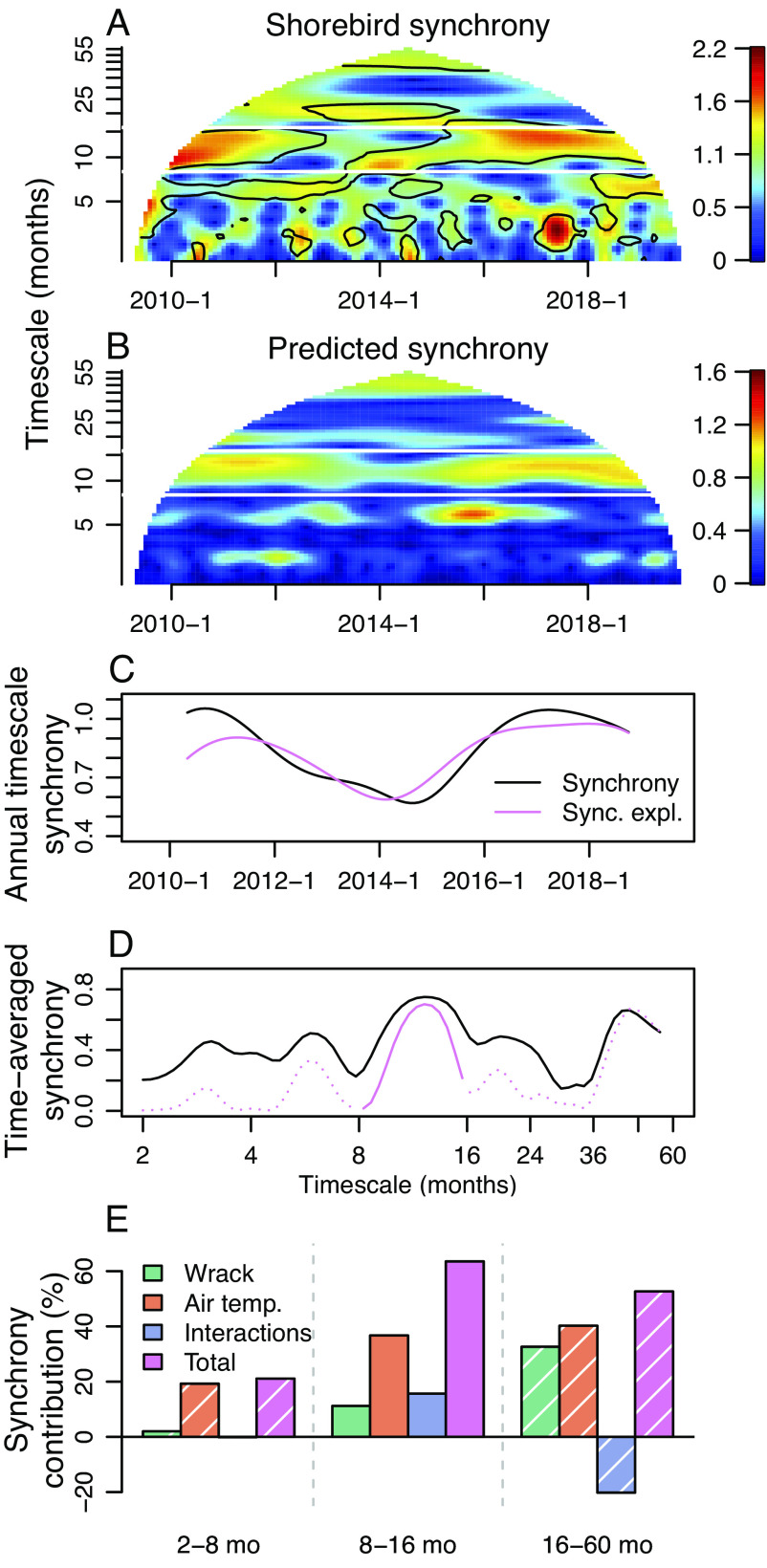
Spatial synchrony in shorebird abundances is largely explained by synchrony in wrack cover and air temperature. (*A*) Time and timescale-specific synchrony in shorebird abundances depicted by the wavelet mean field. Black contours indicate statistically significant synchrony as determined from the wavelet phasor mean field. (*B*) Wavelet-linear-model-predicted shorebird synchrony resembles empirical shorebird synchrony on some timescale bands. (*C*) Isolating the wavelet component at ≈12 mo, predicted synchrony tracks observed synchrony through time. (*D*) The model explains substantial fractions of time-averaged spatial synchrony, especially at 8 to 16 mo timescales. (*E*) Fractions of synchrony in shorebird abundances explained by wrack cover, air temperature, and two-way interaction effects. Portions of (*D*) and (*E*) are dashed and hatched, respectively, since wrack was not significantly coherent with shorebird abundance at 2 to 8 and 16 to 60 mo timescales.

Corroborating the ecosystem-wide consequences of spatially synchronous kelp wrack subsidies, synchrony in kelp wrack cascaded up the beach food web to produce synchrony in the local abundance of predatory shorebirds observed foraging on beaches (Fig. 2). The local abundances of wintering shorebirds on sandy beaches were significantly spatially coherent (P=0.033) with kelp wrack on 8 to 16 mo timescales, with a phase relationship indicating that shorebird abundance tends to lag wrack deposition slightly (ϕ=−0.31). At these timescales, the mechanism underlying the observed pattern is a behavioral response in which birds spend more time in places with more prey availability, not that there is a demographic response as has often been the focus of studies of population spatial synchrony (4). Using wavelet linear modeling, we found that a model with wrack cover and air temperature as predictors explained a large fraction of synchrony in shorebird abundance, though results for 2 to 8 and 16 to 60 mo timescales are taken cautiously since significant coherence occurred only in the 8 to 16 mo timescale band. Air temperature accounts for seasonal cycles in bird abundance associated with their migrations and can influence migration timing (38, 39), and it was added to the model because it improved model diagnostics testing assumptions underpinning quantification of synchrony explained and contributions of driver variables to synchrony.

## Discussion

**Fig. 3.**
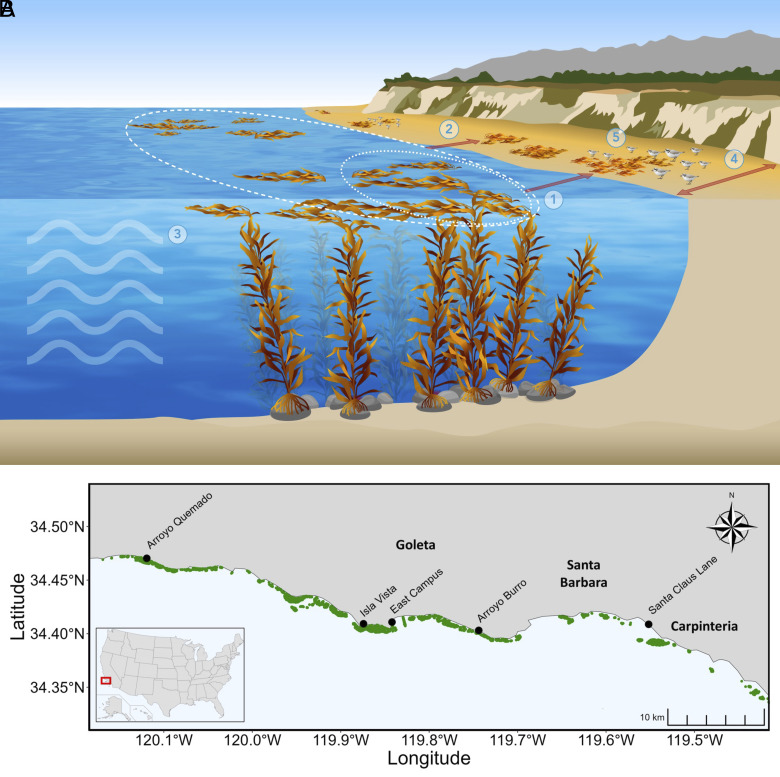
(*A*) Illustration of mechanisms of resource subsidies from kelp forests to sandy beach ecosystems. 1) Local and 2) regional kelp production provide sources of wrack; 3) waves fragment and dislodge kelp, then currents and waves transport drift kelp and deliver it as wrack to beaches; 4) beach width mediates the receptiveness to wrack deposition and wrack retention; 5) wrack on beaches mediates predator (shorebird) abundance by subsidizing the intertidal prey community. Our results provide evidence that these processes occur synchronously across our study sites, producing a cascade of spatial synchrony from kelp forests to sandy beach ecosystems. (*B*) Map of region and study sites. Green-shaded areas represent locations where kelp forest canopy was observed via Landsat during the period 2015 to 2021. Illustration (*A*) by Monica Pessino, Ocean o’ Graphics, UC Santa Barbara.

Our results show that spatial synchrony can cascade across ecosystem boundaries and up food webs via resource subsidies. Spatially synchronous giant kelp biomass fluctuations on shallow rocky reefs provided spatially synchronous resource subsidies to beaches, and those subsidies propagated through beach food webs to produce spatial synchrony in local abundances of wintering shorebirds, an important secondary consumer group (Fig. 3*A*). Shared climatic fluctuations can also synchronize different ecosystems (40–42), but the mechanism we document here is distinct because cross-ecosystem resource subsidies are the synchronizing agent, as opposed to climate synchronizing local production across ecosystems. Generalizing from our results, we should expect that resource subsidies induce cascades of synchrony across ecosystem boundaries when production and transport processes are both synchronized across space, and when the resource subsidy is large relative to other sources. Considering that cross-ecosystem subsidies are widespread and their magnitude can rival or exceed in situ production (1), and that spatial synchrony is extremely common in population dynamics (4) as well as in key ecosystem variables such as primary production (5, 20, 42) and macronutrient concentrations (20, 21, 43), we hypothesize that phenomena of the type we have demonstrated are common.

The delivery of wrack subsidies to beaches is a function of beach proximity to and transport (e.g., wind, currents, waves) from the source ecosystem (44) as well as the driving factors directly affecting both the donor ecosystem (i.e., kelp life history, disturbance) and the recipient ecosystem (i.e., beach morphodynamics) (31, 45). While prior studies also document aspects of this system, our findings integrate these relationships to explain an emergent spatiotemporal property of ecosystem dynamics, synchrony. Kelp wrack subsidies exhibited synchrony across beaches at sub-annual (2 to 8 mo), annual (8 to 16 mo), and interannual (16 to 60 mo) timescales. We identified important drivers of wrack synchrony in both the donor and the recipient ecosystems. Regional, but not local, live kelp biomass was important across timescale bands, suggesting that kelp detritus is transported over larger spatial scales than were considered local in this study (1.5-km radius). Wave height first differences—which correspond to the destructive potential of waves for kelp, (28, 29)—were significantly coherent with wrack time series on sub-annual and annual timescales. Wrack deposition tends to increase with wave action; yet, greater wave intensity is needed to dislodge more strongly attached and deeper water kelp and live kelp biomass is not replenished until new growth occurs the following spring. Episodes of higher wave energy, however, can also cause beach erosion and loss of deposited material. Beach width represents a beach’s receptiveness to wrack subsidies, which can vary seasonally and across tidal cycles, corresponding to both the area available for kelp wrack deposition and the ability to retain wrack subsidies (23). Here, beach width was a significant predictor in wavelet models across all three timescale bands.

The local abundances of shorebirds were coherent with and slightly temporally lagged relative to wrack cover on annual timescales, reflecting a behavioral response to spatiotemporal variation in the availability of wrack-associated invertebrate prey. Understanding relationships between wrack subsidies, beach condition, and shorebirds is of growing importance due to increasing impacts of sea level rise, intensifying coastal development and beach management, and other disturbances (46). Since many shorebird species overwinter in the region (47), the relationship between shorebirds and wrack on annual timescales is significant because it supports the likely strong link between beach condition and shorebird dynamics (17, 24). This result highlights the multitrophic impacts of this cross-ecosystem subsidy where energy derived from wrack inputs not only fuels primary consumer populations but propagates up the food web to secondary consumers. This link to higher trophic levels and more mobile consumers demonstrates how ecosystem subsidies may cross multiple ecosystem boundaries, spanning great distances (48).

Some view studies of spatial synchrony as referring to datasets and analyses where one can examine the spatial extent of synchrony, most commonly done by looking at declines with distance in synchrony (49). However, recent work has instead separated a detailed geographic approach which extends the spatial aspects (5, 22) from an alternative and differently illuminating approach that ignores the spatial/geographic aspects and utilizes timescale structure to carry out inferences instead (6, 21, 27, 33, 50, 51). This study is in the second tradition, in part because inherent logistical constraints on field sampling limited the geographic scope of the study. As such, our study might be considered a proof of concept. Frequent, high spatial resolution satellite imagery could facilitate more geographically extensive observations of beach wrack, allowing examination of the distance-decay and other geographic patterns in this phenomenon, including the extent to which it occurs in other areas. It may be possible to derive wrack data from satellite imagery in future work.

In the context of global climate change, our findings raise critical questions about the importance of synchronous resource subsidies for the long-term functioning of ecosystems, especially those like sandy beaches that depend on resource subsidies. Because synchrony is generally a destabilizing force and the spatial synchrony of environmental drivers may be increasing due to climate change (42, 52), the instability of spatially coupled ecosystems may also be on the rise. At the same time, many ecosystems depend on subsidies from other ecosystems (1, 53, 54). Processes that alter the transport mechanisms responsible for these subsidies could have major impacts on the structure and function of both donor and recipient ecosystems (55, 56). Here, sea level rise, coastal development, and erosion may reduce wrack deposition by limiting beach area and therefore wrack receptivity (32); ocean warming will likely reduce kelp production (57, 58); and increased storm intensity could have complex effects on beach erosion and wrack retention (increasingly reflective shoreline) and removal of kelp plants via waves (28, 29, 59). These potential disruptors to connectivity, and therefore to cross-ecosystem subsidies, are spatially and temporally scale-dependent; while local wave disturbances might disrupt reef-beach connectivity for months, a large-scale marine heatwave or erosion event could disrupt these important linkages for years. Thus, the consequences of climate change for the magnitude and spatial synchrony of cross-ecosystem subsidies could have substantial impacts across ecosystem boundaries and throughout the food web.

## Materials and Methods

### Study System.

The Santa Barbara Channel is a highly productive coastal region with nearshore rocky reef habitat dominated the foundation species giant kelp (*Macrocystis pyrifera*). This region has mixed coastline types but is largely rocky intertidal and sandy beach. Because of the strong ecological connectivity between kelp forests and sandy beaches (17, 18, 23), sandy beach monitoring is conducted at five study sites within the Santa Barbara Channel as part of the Santa Barbara Coastal Long Term Ecological Research (SBC LTER) program (Fig. 3*B*). These sites have been surveyed monthly since 2009, with the exception of April to June 2020, and include Arroyo Quemado (34${}^{{}^{\circ}}$28’13.3”N, 120${}^{{}^{\circ}}$07’09.6”W), Isla Vista (34${}^{{}^{\circ}}$24’33.4”N, 119${}^{{}^{\circ}}$52’27.0”W), East Campus (34${}^{{}^{\circ}}$24’38.6”N, 119${}^{{}^{\circ}}$50’31.3”W), Arroyo Burro (34${}^{{}^{\circ}}$24’11.0”N, 119${}^{{}^{\circ}}$44’38.3”W), and Santa Claus Lane (34${}^{{}^{\circ}}$24’31.4”N, 119${}^{{}^{\circ}}$33’06.7”W).

### Datasets.

Surveys of giant kelp wrack abundance (cover) and dry beach width at the five study sites are conducted monthly at low tide (≤ 0.76 m) along three permanent cross-shore transects. Wrack cover is measured as the total length of giant kelp intersecting the measurement transect for a 1 m wide band from the back beach boundary (i.e., cliff base) to the upper swash limit (17). Wrack is classified as fresh or old and is identified by part of the kelp plant (blade, stipe, or holdfast). We focus our analyses on total blade and stipe wrack because blades and stipes comprise the majority of wrack and are consumed at far higher rates than holdfasts. Wrack cover along replicate transects was averaged by site for each monthly survey. Dry beach width is measured along each transect as the distance from the back beach boundary to the 24-h high tide strandline. The dry beach width measurement is representative of the available beach area for wrack deposition and retention. We used monthly giant kelp wrack data and associated dry beach width data for the period 2009 to 2019 (60).

Data on the biomass of live giant kelp are derived from Landsat satellite imagery using a spectral unmixing algorithm and an empirical relationship relating kelp canopy cover to biomass (34, 35). We obtained monthly data at the spatial resolution of 100 m coastline segments and spatially aggregated the data at two spatial scales: “local,” i.e., averaged within a 1.5-km radius of the beach site, and “regional,” i.e., averaged over all locations along the mainland coast from Point Conception to Carpenteria and San Miguel, Santa Rosa, and Santa Cruz islands (approximately 280 km coastline).

Data on daily maximum significant wave heights corresponding to each beach site were obtained from the CDIP MOP v1.1 model (http://cdip.ucsd.edu/MOP_v1.1/) and were aggregated to a monthly time step, matching the beach wrack data, by averaging. In brief, the model combines hourly empirical wave height and direction measurements with swell propagation and hindcast models (61). Detailed methods for computation of the wave intensity data are given in ref. 37. We analyzed first-differenced time series of wave intensity to better correspond to the mechanisms linking wave intensity to kelp wrack. Following a summer peak in kelp biomass, wave intensity tends to increase through the fall and winter. Initially, relatively small increases in wave intensity can dislodge kelp leading to an increase in wrack; however, once waves have begun to dislodge kelp increasingly strong waves are required to further dislodge kelp and increase wrack.

Data on the abundances of shorebirds at the same sites are collected concurrently with wrack sampling (60). Observers identify (generally, to species) and count the number of birds on 1 km along-shore transects. We considered shorebirds to include American Avocet, Baird’s Sandpiper, Black-bellied Plover, Black-necked Stilt, Black Turnstone, unidentified Dowitchers, Dunlin, Greater Yellowlegs, Killdeer, Least Sandpiper, Lesser Yellowlegs, Long-billed Curlew, Long-billed Dowitcher, Marbled Godwit, Pectoral Sandpiper, Red-necked Phalarope, Red Knot, Ruddy Turnstone, Sanderling, Semipalmated Plover, Short-billed Dowitcher, Snowy Plover, Spotted Sandpiper, Surfbird, Wandering Tattler, Western Sandpiper, Whimbrel, Willet, and Wilson’s Plover. We totaled the abundances of all shorebird species to obtain one aggregate shorebird abundance for each beach and survey event.

Data on monthly average daily air temperatures were obtained at the 5 beach sampling locations from the PRISM dataset (62). Air temperature effectively accounts for seasonal variation in shorebird abundances associated with their seasonal migrations; additionally, local variations in bird migration timing are associated with air temperature (38, 39).

### Analyses.

We quantified spatial synchrony in kelp wrack using wavelet mean fields, which assess spatial synchrony over all sites as a function of time and timescale (27). Statistical significance of spatial synchrony was determined using wavelet phasor mean fields, a closely related technique for which a null model is available, enabling significance testing (27). Statistical significance was assessed relative to 1,000 sets of random phasors, representing a null hypothesis of no synchrony except that arising by chance. Detailed descriptions of these mean field methods, and other analysis methods, and pedagogical figures illustrating their use, are provided in *SI Appendix*.

To examine the synchronizing roles of kelp biomass, waves, and beach width, we used spatial wavelet coherence (27) and wavelet linear models (33). Spatial wavelet coherence tests timescale-specific bivariate relationships between two spatiotemporal variables, giving, for each timescale of interest, a magnitude corresponding to the strength of relationship between the two variables and a phase indicating the temporal offset between peaks in the oscillations of the two variables occurring on the timescale (27). When one variable is an external environmental driver and the other is a biological variable, a statistically significant spatial wavelet coherence relationship indicates that the environmental driver, or possibly some third variable related to both the environmental driver and biological response, is a mechanism of synchrony in the biological response (27). We tested for bivariate spatial wavelet coherences between kelp wrack and local kelp biomass, channel-wide kelp biomass, waves, and beach width (4 drivers × 3 timescale bands = 12 tests). To infer how synchronizing mechanisms change across timescales, we tested for coherence on 2 to 8, 8 to 16, and 16 to 60 mo timescale bands. Significance testing was performed using the “fast” method introduced by Sheppard et al. (50), with 10,000 surrogate (i.e., appropriately randomized) datasets used to test for significance. Because multiple factors drive kelp wrack subsidies, we followed the methods of Sheppard et al. (33) in constructing multivariate wavelet linear models using the combination of all candidate environmental drivers shown to be coherent (P<0.1) with kelp wrack, individually for each timescale band. If both local and channel-wide kelp biomass were coherent with kelp wrack, the one with the lower P-value was selected for the multivariate model. We then applied the wavelet Moran theorem and synchrony attribution theorem of ref. 33 to estimate the fraction of synchrony in kelp wrack explained by these models at a given timescale band.

To test whether synchrony in kelp wrack cascades across trophic levels to produce spatial synchrony in the abundance of shorebirds, we tested for spatial wavelet coherence between shorebird abundance and wrack cover (3 timescale bands = 3 tests). We subsequently applied the wavelet Moran and synchrony attribution theorems to wavelet linear models that included air temperature and wrack cover as predictors. Air temperature was included as a predictor in the wavelet linear model to account for the seasonal cycle in bird abundances arising from their migrations, which improved model diagnostics testing assumptions underpinning quantification of synchrony explained and contributions of driver variables to synchrony. All computations were done in R, using the *wsyn* package, available on the Comprehensive R Archive Network.

Given the potential for multiple testing to produce false positives, we report (above) the numbers of significance tests performed, in order to aid interpretation of strength of evidence for our conclusions. For instance, of the 12 tests in Table 1, a type I error rate of 0.05 would tend to yield 0 or 1 false positives if the null hypothesis of no relationship between variables were true; instead, we observed 7 significant results using a threshold of 0.05, a substantially greater number. Note that our P-values are additionally conservative because they assume that the power spectrum (timescale structure) of response and driver variables arise independently, though in a true driver–response relationship, the driver would, under reasonable linearity assumptions, make the power spectrum of the response variable more similar to its own (51).

## Supplementary Material

Appendix 01 (PDF)

## Data Availability

Previously published data were used for this work (63–65).
