# Supplementary material for: Spatial synchrony cascades across ecosystem boundaries and up food webs via resource subsidies
Source: Proc Natl Acad Sci U S A. 2024 Jan 2;121(2):e2310052120. doi: 10.1073/pnas.2310052120 (PMC10786303; doi:10.1073/pnas.2310052120)
Supplement: Supplementary file 1 — Appendix 01 (PDF) [file pnas.2310052120.sapp.pdf]

Supporting Information for

Spatial synchrony cascades across ecosystem boundaries and up food webs via resource subsidies

Jonathan A. Walter, Kyle A. Emery, Jenifer E. Dugan, David M. Hubbard, Tom W. Bell, Lawrence W. Sheppard, Vadim A. Karatayev, Kyle C. Cavanaugh, Daniel C. Reuman, and Max C.N. Castorani

Jonathan Walter.
E-mail: jawalter@ucdavis.edu
Daniel Reuman.
E-mail: reuman@ku.edu

This PDF file includes:

Supporting text
Figs. S1 to S5
SI References

Supporting Information Text

Details of analysis methods

Wavelet mean field and wavelet phasor mean field. We used wavelet mean fields (1) to quantify the strength of spatial synchrony through time and across timescales, and the closely related wavelet phasor mean field (2) to determine statistical significance. The wavelet mean field is a normalized mean of wavelet transforms of individual time series in a set, the magnitude of which quantifies the strength of synchrony as a function of time and timescale. If $x_n(t)$, for $t = 1, ..., T$, is the nth in a set of time series, then its wavelet transform at timescale (period) σ and time t is denoted $W_{n,\sigma}(t)$. We use the continuous complex Morlet wavelet transform. The wavelet mean field is then defined as

$$r_\sigma(t) = (1/N)\sum_{n=1}^{N} w_{n,\sigma}(t), \quad [1]$$

where

$$w_{n,\sigma}(t) = W_{n,\sigma}(t)/\sqrt{(1/NT)\sum_{n=1}^{N}\sum_{t=1}^{T} W_{n,\sigma}(t)\overline{W_{n,\sigma}(t)}}. \quad [2]$$

Here, overbar denotes complex conjugation. The $w_{n,\sigma}(t)$ are power-normalized transforms that contain essentially the same information as the $W_{n,\sigma}(t)$, particularly as their phases are the same. Further mathematical details are provided in (1).

The magnitude of the wavelet mean field makes sense as a time- and timescale-specific measure of the strength of synchrony because when oscillations at time t and timescale σ are synchronized (i.e., have similar phases in all time series), the magnitude of the wavelet mean field will be large (the sum $\sum_{n=1}^{N} w_{n,\sigma}(t)$ will be a large complex number), whereas unrelated phases will tend to produce a smaller magnitude. Note that due to the normalization in eqn.[2], unlike standard correlation measures, values of the wavelet mean field can exceed 1, but at any timescale the average over time of the wavelet mean field is constrained to the interval 0, 1 inclusive. See Fig. S1 for a demonstration of quantifying timescale-specific, time-localized synchrony using the wavelet mean field.

Whereas the wavelet mean field is sensitive to differences in the magnitude of oscillations (wavelet power), and so is generally preferred for analyses of synchrony, the wavelet phasor mean field quantifies phase alignment only, irrespective of differences in oscillation magnitude. The equation is the same as for the wavelet mean field, except the $w_{n,\sigma}(t)$ are now phasor-normalized, i.e., $w_{n,\sigma}(t) = W_{n,\sigma}(t)/|W_{n,\sigma}(t)|$, where || indicates taking the modulus. The primary benefit for our purposes of the wavelet phasor mean field is that it permits a straightforward significance test against a null model of no synchrony represented by the phase synchrony of n random phasors, where n is the number of time series (here, locations). A distribution of 1000 surrogates representing the null model was generated, and values of the wavelet phasor mean field exceeding the 95th percentile of the surrogate distribution were considered statistically significant.

Spatial wavelet coherence. Spatial wavelet coherence (1) quantifies timescale-specific pairwise relationships between two spatio-temporal variables, extending standard wavelet coherence. Two variables are spatially coherent at a given timescale if they have correlated magnitudes of oscillation and consistent phase differences through time and across locations at that timescale. When one variable is a biotic variable and the other is an external driver (e.g., climate) variable, spatial wavelet coherence can often be used to infer whether the external driver (or some third variable closely related to the external driver variable) is a mechanism of synchrony in the biotic variable. We used spatial wavelet coherence to aid in selecting, from a set of candidate predictors, a more parsimonious set for inclusion in multivariate wavelet linear models (see below). Spatial wavelet coherence between a biological variable, $x_n^{(b)}(t)$, and and environmental variable, $x_n^{(e)}(t)$, both measured at locations $n = 1, \ldots, N$ and times $t = 1, \ldots, T$, is the magnitude of the quantity

$$\Pi_\sigma^{(be)} = (1/NT)\sum_{n=1}^{N}\sum_{t=1}^{T} w_{n,\sigma}^{(b)}(t)\overline{w_{n,\sigma}^{(e)}(t)}, \quad [3]$$

where the $w_{n,\sigma}^{(b)}(t)$ and $w_{n,\sigma}^{(e)}(t)$ are power-normalized wavelet transforms, defined as in eqn.[2] above. The spatial wavelet coherence takes values between 0 and 1, indicating the strength of relationship between the variables (whether they are in phase or phase shifted). The phase relationship between the variables can be extracted from the phase of eqn.[3].

We significance tested spatial wavelet coherence, against a null model of no coherence that preserves the spatial autocorrelation and spectral properties of the empirical data but in which coherence between the variables arises only by chance. We use the fast significance testing procedure introduced by Sheppard *et al* (3), which is conceptually equivalent to the original procedure introduced in (1) but speeds computation and may increase statistical power (3) by keeping all computations in the frequency domain. We summarize here the idea behind original, slower procedure because it is more straightforwardly understood and its essential logic extends to the fast testing procedure. The fast Fourier transform (FFT) is used to produce a frequency-domain representation of the original time series. The phases of the Fourier components are then randomized by imposing random phase perturbations. The phase perturbations used are independent across timescales but identical across locations, thus preserving the spectral properties and spatial autocorrelation of the original time series, but breaking any systematic relationships between temporal oscillations in the two variables, so that any coherence between the randomized variables occurs by chance alone.

The phase-randomized Fourier series are then inverse-transformed back into time series and their spatial coherence quantified. This process is repeated many times to create a distribution of surrograte coherences representing the null hypothesis, and the quantile of the empirical coherence in the distribution of surrogates indicates the strength of evidence that coherence is greater than expected by chance. The quantile can be interpreted as a p-value, specifically, $p = 1 - q$, where q is the quantile of the empirical coherence in the distribution of surrogates. The fast significance testing procedure (3) introduces a stationarity assumption that facilitates quantifying coherence in the frequency domain (i.e., using the Fourier series directly) instead of inverse-transforming back into time series, which is computationally intensive. See Fig. S2 for a demonstration of testing associations between two spatially replicated variables using spatial wavelet coherence. A method of aggregating across timescale bands to produce a single p-value for a whole band, described in (1), is illustrated in Fig. S2, as well.

Note that our significance testing procedures yield a conservative test for coherence (4) because they assume that the power spectra of the two variables arise entirely independently and preserve them in the surrogates representing the null hypothesis. In a true driver-response relationship, under reasonably linearity assumptions, the driver variable will influence the power spectrum of the response variable, making it more similar to that of the driver on timescales where the driver is acting. However, it is not obvious (and is likely highly case-dependent) by how much the power spectrum of the driver affects that of the response, preventing development of a general null model that accounts for this. Methods for developing surrogates that do not preserve power spectra (e.g., white noise time series) are unrealistic and yield unacceptably liberal significance tests, so we use our conservative approach.

Multivariate wavelet linear models. Multivariate wavelet linear models extend spatial wavelet coherence to evaluate the combined effects of multiple predictor variables on a response variable (5). This is analogous to the difference between performing linear regression with a single predictor versus multiple linear regression.

If, adopting notation consistent with the above, $x_n^{(k)}(t)$ for $k = 0, ..., K$ are spatio-temporal variables measured at times $t = 1, ..., T$ and locations $n = 1, ..., N$; and if $w_{n,\sigma}^{(k)}(t)$ are their power-normalized (eqn. [2]) wavelet transforms, then wavelet linear models statistically explain variation in the response transforms $w_{n,\sigma}^{(0)}(t)$ in terms of the predictor transforms $w_{n,\sigma}^{(k)}(t)$:

$$w_{n,\sigma}^{(0)}(t) \approx \beta_1(\sigma) w_{n,\sigma}^{(1)}(t) + ... + \beta_K(\sigma) w_{n,\sigma}^{(K)}(t). \quad [4]$$

The $\beta_k(\sigma)$ are complex-valued coefficients which can depend on timescale (σ). The fitted coefficients maximize the spatial coherence between the left and right sides of eqn. [4]. See (5) for the full theoretical development of this modelling framework.

Synchrony attribution and synchrony explained. Sheppard et al. (1) developed a "wavelet Moran theorem," that extended to a wavelet context the observation by Moran (6) that, assuming identical linear population dynamics, the synchrony (correlation) between two populations equals the synchrony in environmental conditions. This was extended first to a spatial wavelet coherence context (1), allowing timescale-specific estimation of the synchrony in an ecological variable that can be explained by a single environmental driver variable; and subsequently to a multivariate wavelet linear model context (5). Below, we summarize the main results, referring the reader to (1) and (5) for all details. Additionally, all wavelet models were analyzed using the *wsyn* package in R, which is available on the Comprehensive R Archive Network. The package includes a "vignette" which explains how wavelet models are fitted and analyzed using the software, and how to interpret results. Interactions between Moran effects of distinct drivers are part of the theory. Pedagogical examples illustrating how drivers can interact synergistically or antagonistically are shown in Figs. S3 and S4, respectively. See also (7) for a comprehensive analytic version of the theory of interacting Moran effects.

Let $v_{n,\sigma}(t) = \beta_1(\sigma) w_{n,\sigma}^{(1)}(t) + ... + \beta_K(\sigma) w_{n,\sigma}^{(K)}(t)$, i.e., the right-hand side of eqn. 4, which is the wavelet model for the transforms $w_{n,\sigma}^{(0)}(t)$. Then define the residuals $d_{n,\sigma}(t) = w_{n,\sigma}^{(0)}(t) - v_{n,\sigma}(t)$. Let $h_{n,\sigma} = \frac{v_{n,\sigma}(t)}{||v_{n,\sigma}(t)||}$. Here, we are considering the $w_{n,\sigma}^{(k)}(t)$, for each fixed k and σ and for $n = 1, \ldots, N$ and t ranging across the times for which $w_{n,\sigma}^{(k)}(t)$ is defined, to be an element of the complex inner-product space $\mathbb{C}^{D_\sigma}$ for D_σ equal to N times the number of distinct t for which $w_{n,\sigma}^{(k)}(t)$ is defined. The norm $|| \cdot ||$ is then the norm based on the standard inner product for that space. We then let $\Pi_\sigma^{(0h)} = ||v_{n,\sigma}(t)||$, so that $w_{n,\sigma}^{(0)}(t) = \Pi_\sigma^{(0h)} h_{n,\sigma}(t) + d_{n,\sigma}(t)$. The authors of reference (5) proved the following theorem, reproduced here without change.

Theorem 1 A Moran theorem for wavelet linear models. *Using the notation defined above, and if* $r_\sigma^{(0)}(t) = \frac{1}{N}\sum_{n=1}^{N} w_{n,\sigma}^{(0)}(t)$ *is the wavelet mean field of the* $x_n^{(0)}(t)$, $r_\sigma^{(h)}(t) = \frac{1}{N}\sum_{n=1}^{N} h_{n,\sigma}(t)$ *and* $r_\sigma^{(d)}(t) = \frac{1}{N}\sum_{n=1}^{N} d_{n,\sigma}(t)$, *then the time-averaged squared magnitude of* $r_\sigma^{(0)}(t)$ *is*

$$\frac{1}{T}\sum_{t=1}^{T} |r_\sigma^{(0)}(t)|^2 = |\Pi_\sigma^{(0h)}|^2 \frac{1}{T}\sum_{t=1}^{T} |r_\sigma^{(h)}(t)|^2 \quad [5]$$

$$+ \frac{1}{TN^2}\sum_{t=1}^{T}\sum_{n=1}^{N}\sum_{m \neq n} 2\,\mathrm{Re}(\Pi_\sigma^{(0h)} h_{n,\sigma}(t) \overline{d_{m,\sigma}(t)}) \quad [6]$$

$$+ \frac{1}{T}\sum_{t=1}^{T} |r_\sigma^{(d)}(t)|^2. \quad [7]$$

If populations $x_n^{(0)}(t)$ do not interact with populations $x_m^{(0)}(t)$ (for $m \neq n$) at neighboring sites, nor are they directly affected by other conditions (environmental or biotic) in neighboring sites, interpreted as the term in (6) being negligible (see justification in the proof of this theorem in Appendix S14 of (5)), then

$$\frac{1}{T}\sum_{t=1}^{T}|r_\sigma^{(0)}(t)|^2 \approx |\Pi_\sigma^{(0h)}|^2 \frac{1}{T}\sum_{t=1}^{T}|r_\sigma^{(h)}(t)|^2 + \frac{1}{T}\sum_{t=1}^{T}|r_\sigma^{(d)}(t)|^2. \quad [8]$$

In other words, synchrony of $x_n^{(0)}(t)$ can be divided into a component attributable to the variables $x_n^{(k)}(t)$, $k = 1, \ldots, K$, and residual synchrony. It follows that

$$\{|r_\sigma^{(0)}(t)|\}_t \geq |\Pi_\sigma^{(0h)}|^2 \{|r_\sigma^{(h)}(t)|\}_t, \quad [9]$$

where $\{x\}_t$ represents the time average of the square of x. If, in addition, the only synchronizing influence on $x_n^{(0)}(t)$ is the variables $x_n^{(k)}(t)$, $k = 1, \ldots, K$, interpreted as the second term on the right of (8) being negligible, then

$$\{|r_\sigma^{(0)}(t)|\}_t \approx |\Pi_\sigma^{(0h)}|^2 \{|r_\sigma^{(h)}(t)|\}_t. \quad [10]$$

Thus $|\Pi_\sigma^{(0h)}|^2\{|r_\sigma^{(h)}(t)|\}_t$ gives the amount of synchrony explained solely by the synchronizing influence of the variables $x_n^{(k)}(t)$, $k = 1, \ldots, K$.

Extending the logic of the wavelet Moran theorem, the proportion of synchrony explained by the model was quantified as

$$q(\sigma) = \frac{|\Pi_\sigma^{(0h)}|^2 \frac{1}{T}\sum_{t=1}^{T}|r_\sigma^{(h)}(t)|^2}{\frac{1}{T}\sum_{t=1}^{T}|r_\sigma^{(0)}(t)|^2}. \quad [11]$$

Above, we made the assumption that (6), corresponding to interactions between sites, is negligible. We tested this assumption directly for our data by examining whether the so-called "cross terms",

$$\frac{\frac{1}{TN^2}\sum_{t=1}^{T}\sum_{n=1}^{N}\sum_{m\neq n} 2\mathrm{Re}(\Pi_\sigma^{(0h)} h_{n,\sigma}(t)\overline{d_{m,\sigma}(t)})}{\frac{1}{T}\sum_{t=1}^{T}|r_\sigma^{(0)}(t)|^2}, \quad [12]$$

when averaged over a timescale band, were small. For our data, cross terms were always $< 10\%$, so our estimates of proportions of synchrony explained were considered adequate.

The framework above is expanded further in the "synchrony attribution theorem" of (5). Using that theorem, proportions of synchrony attributable to individual drivers and interactions between drivers can be computed, as in, for instance, Figs 1e and 2e in the main text. We refer the reader to Appendix S17 of reference (5).

Testing for annual-timescale leakage in the wavelet mean field

To define the annual timescale band, we tested the extent of leakage in the wavelet mean field for a regular annual cycle. Leakage refers to the effect on nearby timescales of regular oscillations at a particular timescale due to the imperfect timescale resolution of wavelet analysis; wavelet analysis trades off timescale resolution to enable localization of dynamics in time. We constructed $n = 5$ 132-time step (matching our empirical data) synthetic time series featuring a regular, period-12 sine wave and Gaussian (white) random noise with mean = 0 and standard deviation = 0.25. Gaussian noise has a flat power spectrum and so makes our synthetic data more similar to empirical time series than a perfectly regular sinusoid, but has no effect on the timescale structure of the data. The wavelet mean field of the synthetic data showed high synchrony at timescales 8-16 (i.e., 12 ± 4) time steps (Fig. S5). This establishes the symmetry of the band. We defined the annual timescale band as spanning 8-16 months, though the width of the band of high synchrony is sensitive to the relative amplitudes of annual cycle versus noise. A lower signal-to-noise ratio would produce a narrower band of high synchrony, so our choice of band width is conservative from the perspective of capturing all the synchrony associated with annual variability. The subannual and interannual timescale bands covered, respectively, the timescales shorter and longer than the annual band. For this and all other analyses, the wavelet shape parameter $f_0 = 1$; note that band width is sensitive to choice of f_0.

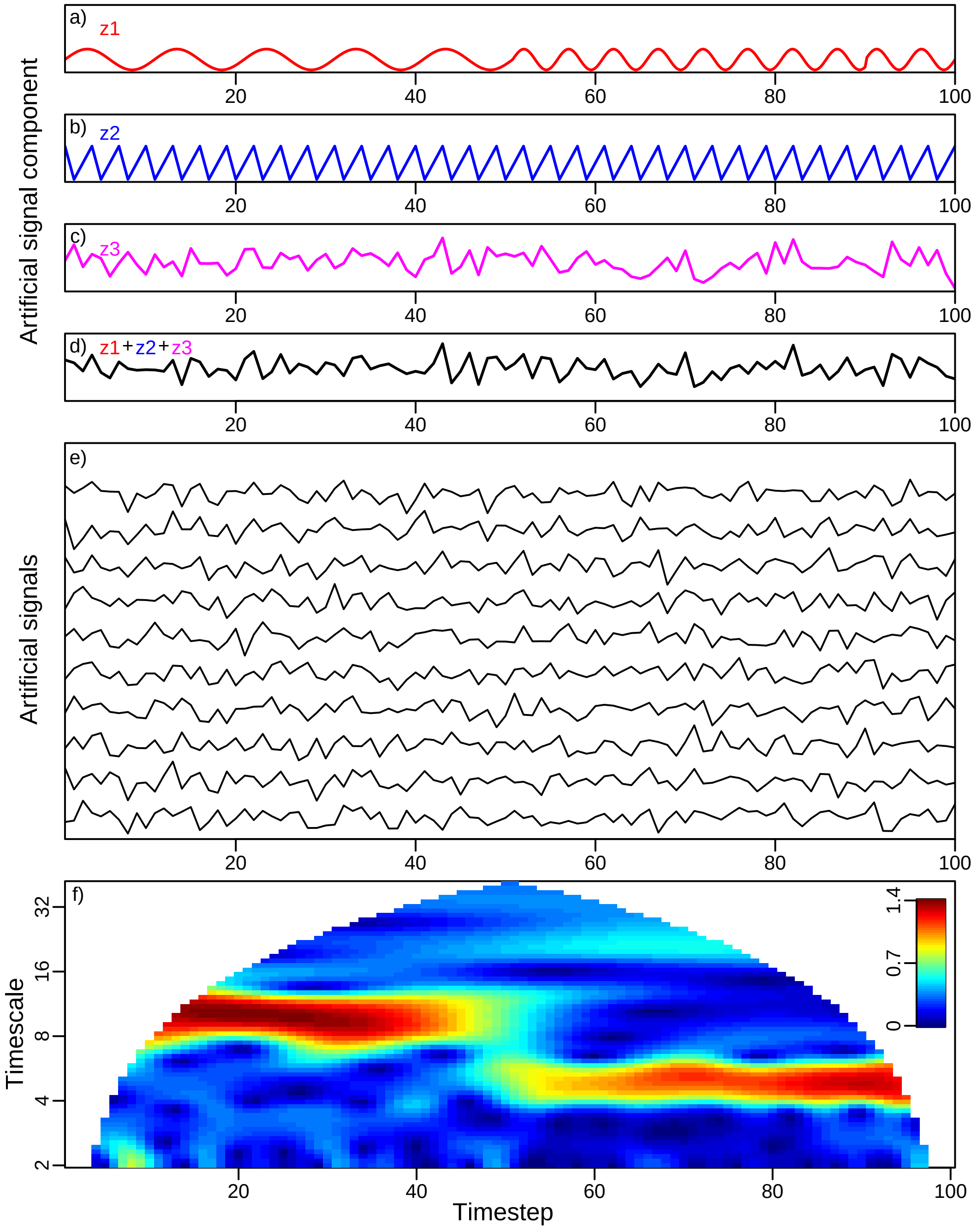

Fig. S1. Measuring timescale-specific, time-localized synchrony using the wavelet mean field. We create 10 artificial signals (time series in e) using components shown in panels (a-c). The signal component in (a) begins as a period-10 sine wave and changes halfway through to a period-5 sine wave. This will be shared across all 10 time series as a synchronous component with changing dominant timescale of synchrony. Panel (b) shows a period-3 triangular wave; this is offset in each signal by a random number of time steps, creating asynchronous but period-3 fluctuations in the resulting time series. Panel (c) is white noise, generated independently for each time series. Panel (d) shows one instance of the sum of the three components. Panel (e) shows 10 instances of their combination, representing 10 different locations; these are not obviously synchronized. Panel (f) displays the wavelet mean field magnitude of the signals in (e). Note that the wavelet mean field recovers the synchronous component depicted in panel (a), but is unaffected by the periodic but asynchronous time series depicted in panel (b). This figure was inspired by and parallels closely Supplementary Figure 1 in (1).

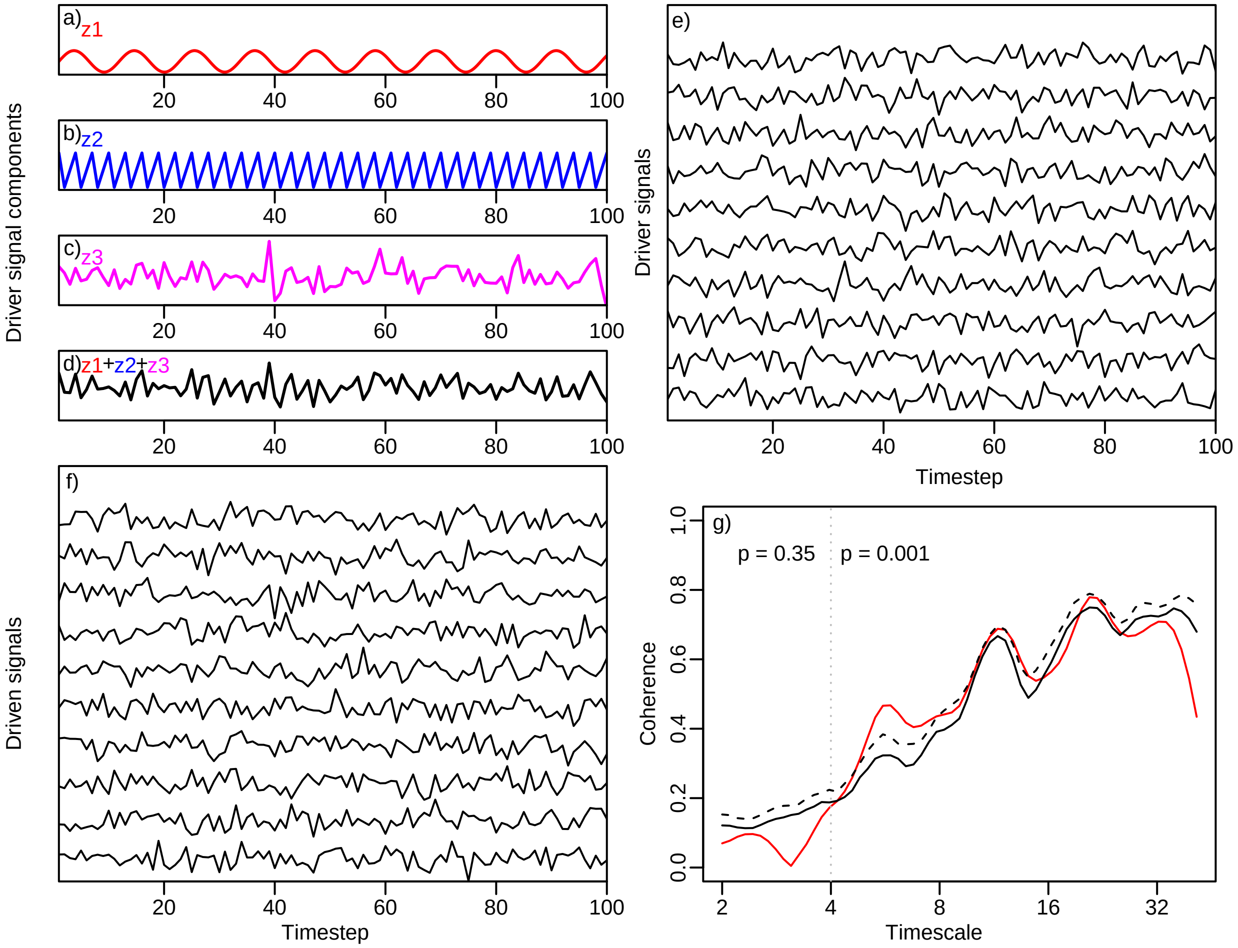

Fig. S2. Measuring timescale-specific associations between variables using spatial wavelet coherence. Similar to Fig. S1, panels (a-c) represent component signals, and panel (d) represents an instance of their combination. However, the component in panel (a) is here a period-11 sine wave. Here, we assume that these are components of an environmental driver variable; panel (e) shows instances of their combination for 10 locations. Unlike in Fig. S1, the component in panel (a) is randomly phase-shifted when creating the signals in panel (e), so that the driver variable is asynchronous. We assume that an ecological phenomenon is driven by the signals in panel e via a moving average process, i.e., the driven signals $y_n(t) = (\sum_{k=0}^{3} x_n(t-k))/3 + \epsilon_n(t)$, where $x_n(t)$ are the driver signals at locations n and times t, and the $\epsilon_n(t)$ are independent, normally distributed noise with mean 0 and standard deviation 1.5. The order of the moving average process means that the component in panel (b) does not translate to the driven variable, but the component in panel (a) does. The driven signals are shown in panel (f). Panel (g) shows the spatial coherence between the driver and driven variable. The red line is the empirical coherence, the solid black line is the 95th percentile of coherences of surrogates, and the dashed black line is the 99th percentile. We use a rank averaging procedure (1) to assign a p-value to the timescale bands 2-4 time steps and >4 time steps. Because the operating driver variable has a period-11 oscillation, long-timescale (>4 time steps) coherence is highly significant, but not at short (2-4 timesteps) timescales. This figure was inspired by and parallels closely Supplementary Figure 5 in (1).

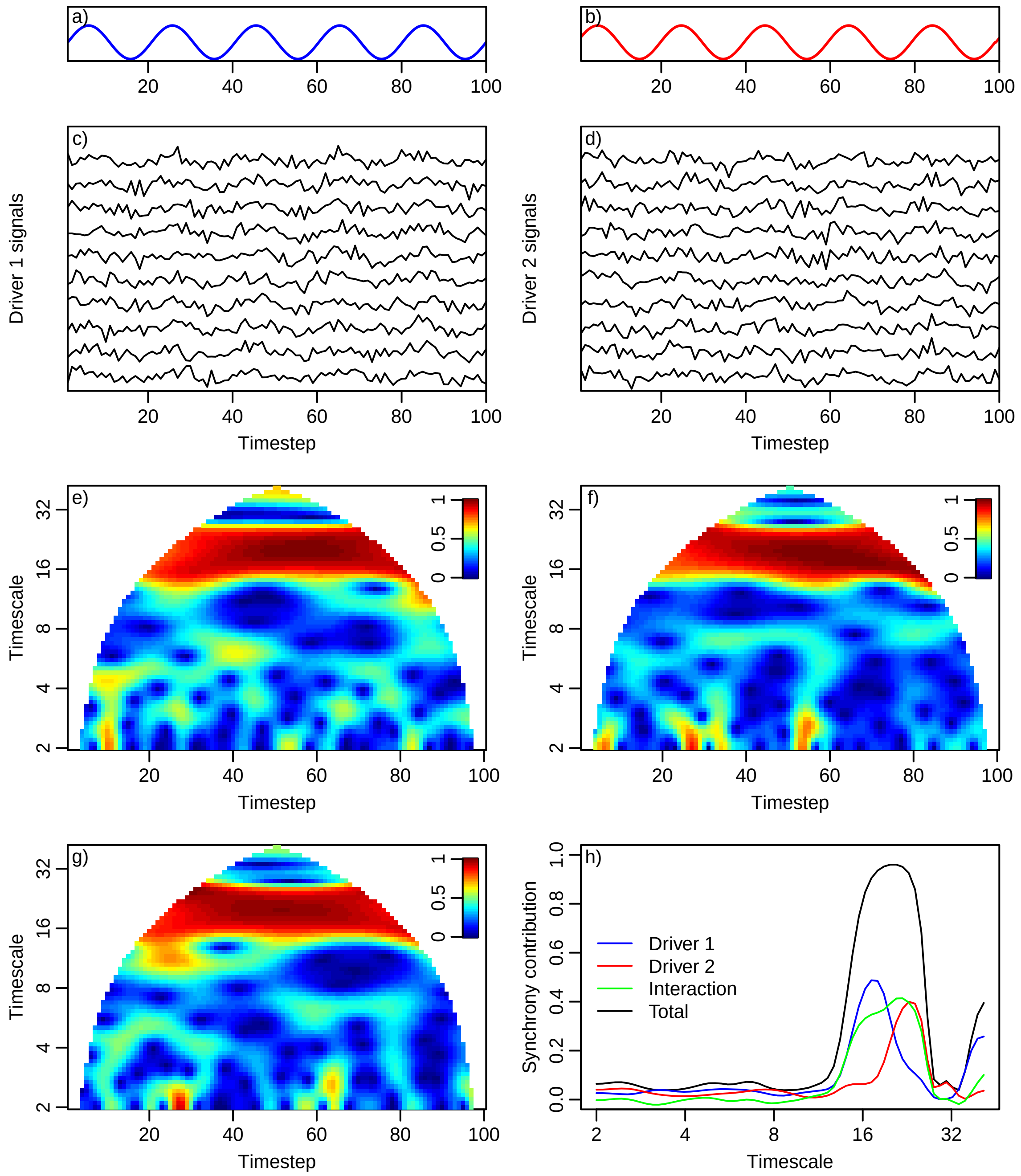

Fig. S3. Multivariate wavelet models with synergistic interaction between drivers. Panels (a-b) show period-20 sine waves with a small phase offset (1 time step) between them. These are underlying synchronous components of driver 1 and driver 2. Panels (c-d) show instances of driver 1 and driver 2 for 10 locations; each signal consists of the period-20 sine wave above it and white noise with standard deviation 1. Panels (e-f) show the corresponding wavelet mean fields of the drivers. Panel (g) shows the wavelet mean field of a hypothetical ecological variable, $y_n(t)$, affected by driver 1 and driver 2: $y_n(t) = x_n^{(1)}(t) + x_n^{(2)}(t) + \epsilon_n(t)$, where the $x_n^{(k)}(t)$ are environmental drivers at location n and time t, and $\epsilon_n(t)$ is independent and normally distributed noise with mean 0 and standard deviation 1. Panel (h) shows the contribution of each driver and their interaction to synchrony in the ecological variable (g) as a function of timescale. Because the phases of the driver variables are nearly aligned and their effects on the ecological variable are both positive and unlagged, their interaction effect is positive (synergistic): total synchrony is greater than either driver acting independently. This figure was inspired by and parallels Figure 1 in (5).

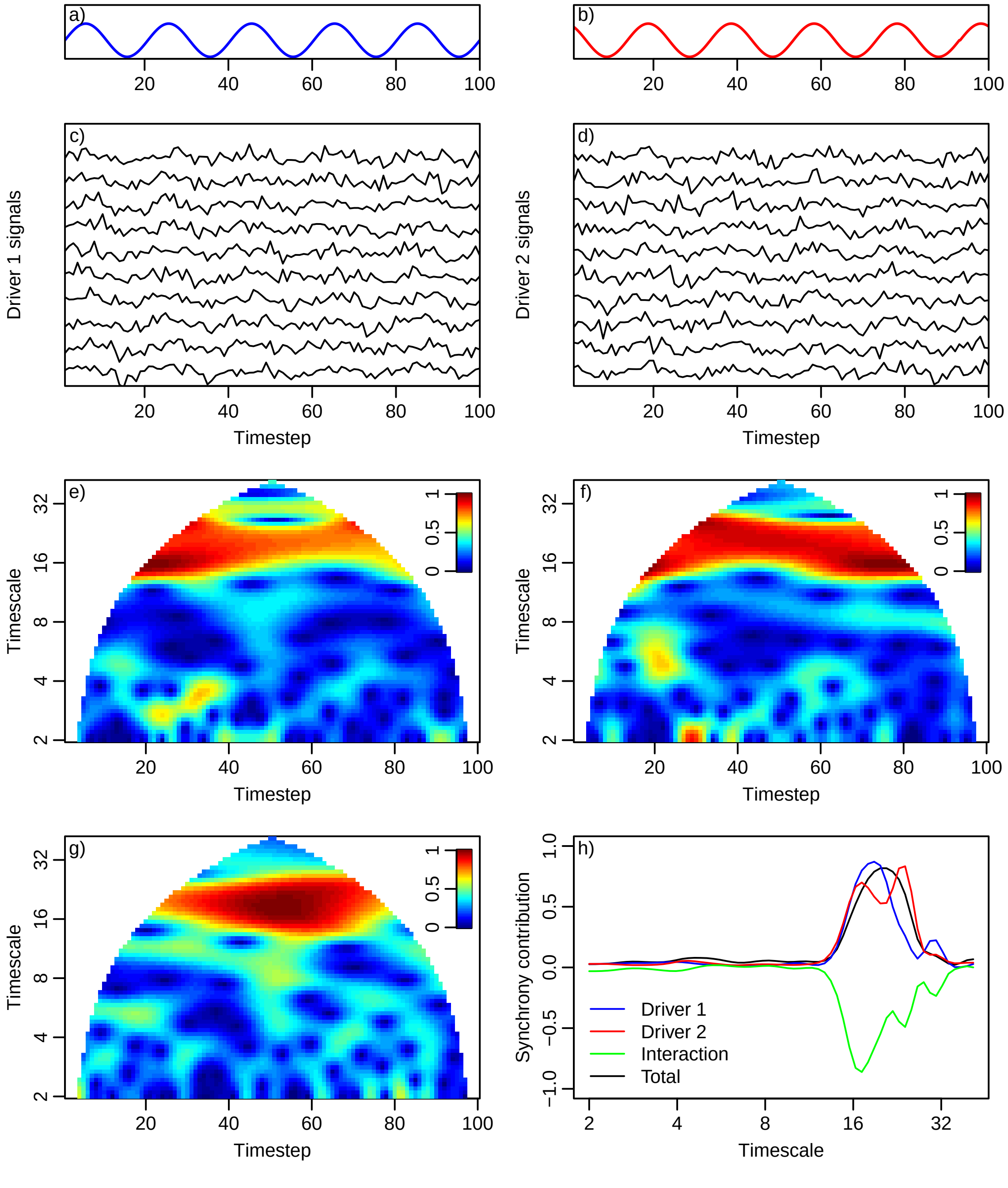

Fig. S4. Multivariate wavelet models with antagonistic interaction between drivers. Panels (a-b) show period-20 sine waves with a large phase offset (7 time steps) between them. These are underlying synchronous components of driver 1 and driver 2. Panels (c-d) show instances of driver 1 and driver 2 for 10 locations; each signal consists of the period-20 sine wave above it and white noise with standard deviation 1. Panels (e-f) show the corresponding wavelet mean fields of the drivers. Panel (g) shows the wavelet mean field of a hypothetical ecological variable, $y_n(t)$, affected by driver 1 and driver 2: $y_n(t) = x_n^{(1)}(t) + x_n^{(2)}(t) + \epsilon_n(t)$, where the $x_n^{(k)}(t)$ are environmental drivers at location n and time t, and $\epsilon_n(t)$ is independent and normally distributed noise with mean 0 and standard deviation 1. Panel (h) shows the contribution of each driver and their interaction to synchrony in the ecological variable (g) as a function of timescale. Because the phases of the driver variables are offset by $> 1/4$ cycle and their effects on the ecological variable are both positive and unlagged, their interaction effect is negative (antagonistic); total synchrony is less than if the drivers each acted independently, and at some timescales the contribution of an individual driver to synchrony exceeds observed synchrony, due to the negative interaction between the drivers.This figure was inspired by and parallels Figure 1 in (5).

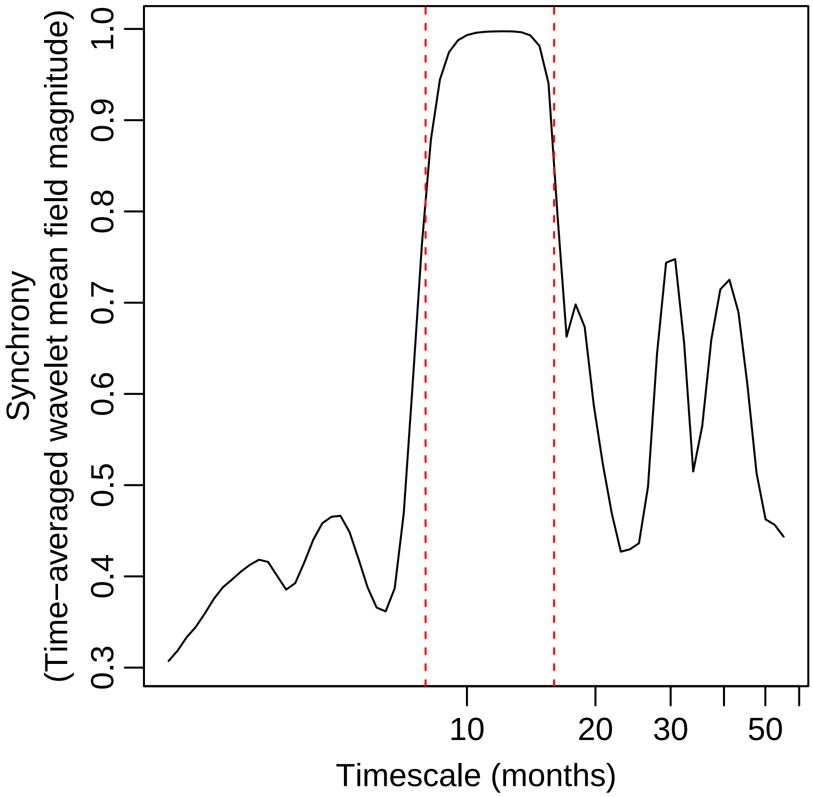

Fig. S5. The time-averaged wavelet mean field magnitude of synthetic time series with a period-12 (annual) cycle. Dashed red lines denote 8- and 16-month timescales, corresponding to our definition of the annual timescale band, given that wavelet analysis has imperfect timescale precision.

References

1. LW Sheppard, JR Bell, R Harrington, DC Reuman, Changes in large-scale climate alter spatial synchrony of aphid pests. *Nat. Clim. Chang.* **6**, 610–613 (2016).
2. TL Anderson, et al., The dependence of synchrony on timescale and geography in freshwater plankton. *Limnol. Oceanogr.* **64**, 483–502 (2019).
3. L Sheppard, P Reid, D Reuman, Rapid surrogate testing of wavelet coherences. *EPJ Nonlinear Biomed. Phys.* **5**, 1 (2017).
4. JA Walter, et al., Weather and regional crop composition variation drive spatial synchrony of lepidopteran agricultural pests. *Ecol. Entomol.* **45**, 573–582 (2020).
5. LW Sheppard, EJ Defriez, PC Reid, DC Reuman, Synchrony is more than its top-down and climatic parts: interacting moran effects on phytoplankton in british seas. *PLoS Comput. Biol.* **15**, e1006744 (2019).
6. PA Moran, The statistical analysis of the canadian lynx cycle. *Aust. J. Zool.* **1**, 291–298 (1953).
7. DC Reuman, et al., How environmental drivers of spatial synchrony interact. *Ecography* p. e06795 (2023).
